# Supplementary material for: HDG-select: A novel GUI based application for gene selection and classification in high dimensional datasets
Source: PLoS One. 2021 Jan 28;16(1):e0246039. doi: 10.1371/journal.pone.0246039 (PMC7842997; doi:10.1371/journal.pone.0246039)
Supplement: S1 Table — (DOCX) [file pone.0246039.s007.docx]

**S1 Table.**

| **Dataset name** | **Original dataset** | **Approach** | **TT filter** | **WRS filter** | **Combined filter** |
| --- | --- | --- | --- | --- | --- |
| Breast | 68.04 | Filter | 78.00 | 81.55 | 79.00 |
|  |  | Filter-GBPSO | 84.44 | **88.55** | 87.44 |
| CNS | 68.33 | Filter | 86.66 | 86.66 | 88.33 |
|  |  | Filter-GBPSO | 95.00 | 91.66 | **100.00** |
| Colon | 87.10 | Filter | 91.66 | 88.33 | 91.66 |
|  |  | Filter-GBPSO | 96.66 | 98.33 | **100.00** |
| Leukemia | 98.61 | Filter | 97.32 | 97.32 | 97.32 |
|  |  | Filter-GBPSO | 98.75 | **100.00** | **100.00** |
| Ovarian | **100.00** | Filter | 100.00 | 100.00 | 100.00 |
|  |  | Filter-GBPOS | **100.00** | **100.00** | **100.00** |
| Prostate | 88.24 | Filter | 96.18 | 96.18 | 96.18 |
|  |  | Filter-GBPSO | **100.00** | **100.00** | 99.00 |
