# Supplementary material for: HDG-select: A novel GUI based application for gene selection and classification in high dimensional datasets
Source: PLoS One. 2021 Jan 28;16(1):e0246039. doi: 10.1371/journal.pone.0246039 (PMC7842997; doi:10.1371/journal.pone.0246039)
Supplement: S2 Table — (DOCX) [file pone.0246039.s008.docx]

| **Dataset name** | **Approach** | **TT filter** | **WRS filter** | **Combined filter** |
| --- | --- | --- | --- | --- |
| Inflammatory Breast Cancer | Filter | 91.7 | 92.00 | 85.00 |
|  | Filter-GBPSO | 96.00 | 98.00 | **98.00** |
| Breast Cancer | Filter | 85 | 85.00 | 92.5 |
|  | Filter-GBPSO | 97.5 | 97.5 | **97.5** |
| Brain Metastatic Breast Cancer | Filter | 100 | 95 | 100 |
|  | Filter-GBPSO | 100.0 | **100.0** | 97.5 |
| Autism | Filter | 86.3 | 83.8 | 86.9 |
|  | Filter-GBPSO | 91.1 | 87.3 | **92.1** |
| Influenza A | Filter | 100 | 100 | 100 |
|  | Filter-GBPOS | **100** | **100** | **100** |

**S2 Table.**
