# Supplementary material for: HDG-select: A novel GUI based application for gene selection and classification in high dimensional datasets
Source: PLoS One. 2021 Jan 28;16(1):e0246039. doi: 10.1371/journal.pone.0246039 (PMC7842997; doi:10.1371/journal.pone.0246039)
Supplement: S3 Table — (DOCX) [file pone.0246039.s009.docx]

**S3 Table.**

| **Dataset name** | **PCC-BPSO-SVM [11]** | **PCC-GA-SVM [11]** | **Best filter-GBPSO-SVM (current work)** | **Combined filter-GBPSO-SVM (current work)** |
| --- | --- | --- | --- | --- |
| Breast | 90.72 | 88.66 | **100.00** | 87.44 |
| CNS | 98.33 | 98.33 | **100.00** | **100.00** |
| Colon | 91.94 | 91.94 | **100.00** | **100.00** |
| Leukaemia | **100.00** | **100.00** | **100.00** | **100.00** |
| Ovarian | **100.00** | **100.00** | **100.00** | **100.00** |
| Prostate | 97.06 | 96.08 | **100.00** | 99.00 |
